# Recent advances in the management of non-small cell lung cancer

**DOI:** 10.12688/f1000research.11471.1

**Published:** 2017-12-07

**Authors:** Samira Shojaee, Patrick Nana-Sinkam

**Affiliations:** 1 Division of Pulmonary and Critical Care Medicine, Virginia Commonwealth University, Richmond, USA

**Keywords:** EGFR, Non-Small Cell Lung Cancer, ALK mutation

## Abstract

Lung cancer is the number one cause of cancer-related death in both men and women. However, over the last few years, we have witnessed improved outcomes that are largely attributable to early detection, increased efforts in tobacco control, improved surgical approaches, and the development of novel targeted therapies. Currently, there are several novel therapies in clinical practice, including those targeting actionable mutations and more recently immunotherapeutic agents. Immunotherapy represents the most significant step forward in eradicating this deadly disease. Given the ever-changing landscape of lung cancer management, here we present an overview of the most recent advances in the management of non-small cell lung cancer.

## Personalized targeted therapy

Over the past decade, the treatment of non-small cell lung cancer (NSCLC) has evolved. While early diagnosis and surgical treatment results in optimal patient outcomes, the majority of patients are diagnosed with lung cancer at later, largely incurable stages often requiring multimodality therapy. Over the last decade, we have observed significant improvements in the management of advanced stages primarily due to an increased understanding of the molecular heterogeneity and drivers of lung cancer initiation and progression as well as improvements in radio and surgical therapies. Using high-throughput platforms, investigators have determined that a significant percentage of lung tumors have actionable somatic mutations, each of which represents an opportunity for novel targeted or “personalized” therapy. Investigators have discovered mutations within the epidermal growth factor receptor (EGFR), anaplastic lymphoma kinase (ALK), fibroblast growth factor receptor (FGFR), ROS-1, KRAS, HER2, BRAF, MET, and RET ^1– 7^. Through clinical trials, distinct roles for targeted therapies are emerging as standard first- or second-line therapies in patients harboring select mutations.

### EGFR mutation

Mutations in EGFR, first discovered in 2004 ^8^, are present in up to 10–15% of all patients with NSCLC ^9^. Initial studies described the most common demographics as never-smokers, female gender, and Asian ethnicity ^9– 12^. Mutations are predominantly located in EGFR exons 18–21. A total of 85% of the mutations are either deletions in exon 19 or a single-point substitution mutation in exon 21 (l858R) ^11, 12^. EGFR tyrosine kinase inhibitors (gefitinib, erlotinib, and afatinib) are the currently available first-line agents for patients harboring EGFR-positive mutations with an estimated initial response rate of 70–80% ^13, 14^. Patients with stage IV NSCLC and EGFR mutations treated with tyrosine kinase inhibitors experienced improved progression-free survival (PFS), increased response rate, better overall quality of life, and fewer side effects in comparison to those receiving standard platinum-based chemotherapy ^15^. The majority of patients, however, experience disease progression secondary to acquired resistance. Up to 60% of acquired resistance mutations are driven by T790M ^16^. T790M is an activating point mutation in exon 20 which substitutes methionine for threonine and interferes with the binding of tyrosine kinase inhibitors ^6^. Next-generation EGFR tyrosine kinase inhibitors targeting T790M mutations have been developed in the event of resistance to first-line therapy ^17^. Jänne and colleagues showed that among those with positive T790M mutations and evidence of disease progression on EGFR tyrosine kinase inhibitors, the use of next-generation tyrosine kinase inhibitors with specific T790M targeting such as osimertinib resulted in a response rate of 61% (95% CI: 52–70) as opposed to a response rate of 21% (95% CI: 12–34) when there was no centrally detectable T790M mutation. The authors reported a median PFS of 9.6 months versus 2.8 months in EGFR-T790M-negative patients ^18– 20^. Osimertinib was approved by the U.S. Food and Drug Administration (FDA) for the treatment of EGFR-T790M-positive NSCLC with disease progression on a prior EGFR tyrosine kinase inhibitor ^20^. The median overall survival (OS) is likely to improve following the approval of third-generation tyrosine kinase inhibitors such as osimertinib, which showed a median PFS of 9.6 months for patients without central nervous system (CNS) metastasis ^21, 22^. Additionally, necitumumab, a recently approved second-generation EGFR monoclonal antibody, showed improved OS in advanced squamous NSCLC when combined with cytotoxic chemotherapy ^20^. In an open-labeled randomized phase III trial in 1,093 patients with stage IV NSCLC, Thatcher *et al.* showed that OS was significantly prolonged in the necitumumab plus gemcitabine and cisplatin group (OS: 11.5 months [95% CI: 10.4–12.6]) versus gemcitabine and cisplatin alone (OS: 9.9 months [95% CI: 8.9–11.1]) ^23^.

### ALK rearrangement

A total of 4–5% of NSCLC adenocarcinomas harbor ALK genetic rearrangements. A short inversion in chromosome 2p results in rearrangement of the receptor tyrosine kinase ALK and fusion of the intracellular kinase domain with the amino acid end of echinoderm microtubule-associated protein like 4 (EML4). Many variants of EML4–ALK fusions have been identified, but most commonly exons 1–13 of EML4 join exons 20–29 of ALK ^7^. EML4–ALK fusion leads to a ligand-independent, constitutive activation of the rearranged ALK receptor, which is responsible for both tumor cell proliferation and survival ^7, 24^. Frequently detected in young never-smoker males ^25^, ALK fusions are thought to occur mutually exclusively of EGFR mutations. ALK fusions are primarily sensitive to the ALK inhibitor crizotinib ^7, 24, 25^. In 2011, the results of 119 ALK-rearranged NSCLC patients were combined with those of 136 ALK-rearranged patients from another trial and formed the efficacy data that led to conditional FDA approval of crizotinib ^26^. A total of 72% of 119 patients were never-smokers and 97% had adenocarcinoma histology. The response rate was 61% with an estimated PFS of 10 months (95% CI: 8.2–14.7). By 2013, a randomized phase III trial (PROFILE-1007) confirmed the efficacy of crizotinib in ALK-rearranged NSCLC ^27– 29^. The majority of patients, however, developed resistance relapsing within 12 months ^30^. Ceritinib, a novel ALK inhibitor, showed greater potency compared to crizotinib in a phase I study of 130 patients. Antitumor activity was independent of prior ALK-inhibitor therapy ^31, 32^. There was an overall response rate of 58% (95% CI: 48–67). The response rate was also reported at 56% (95% CI: 45–67) among those who had previously received crizotinib. Among patients with NSCLC who received at least 400 mg of ceritinib per day, the median PFS was 7.0 months (95% CI: 5.6–9.5). On April 2014, ceritinib received an accelerated FDA approval for use in patients with metastatic ALK-positive NSCLC who were previously treated with crizotinib. Along with ceritinib, alectinib, another FDA-approved ALK inhibitor, has potential advantages over crizotinib including greater specificity, sensitivity, and ability to cross the blood–brain barrier as well as a different spectrum of activity against resistance mutations ^26, 33– 35^. While most patients with ALK- or ROS1-positive NSCLC develop resistance to tyrosine kinase inhibitor therapy, loratinib, a selective brain-penetrant ALK/ROS1 tyrosine kinase inhibitor, is active against most known resistance mutations and has been granted FDA breakthrough therapy status. In a study of 54 patients with ALK- or ROS1-positive NSCLC with or without brain metastases, loratinib treatment resulted in durable clinical responses with a 50% overall response rate, including intracranial responses, in ALK- and ROS1-positive NSCLC patients, many of whom had CNS metastases. Nearly 50% of the patients had prior tyrosine kinase inhibitor therapy and 39 patients had CNS metastasis ^35, 36^.

### KRAS and MEK mutation

Kirsten rat sarcoma viral oncogene ( *KRAS*), a RAS proto-oncogene, has a critical role in signal transduction pathways that regulate cell proliferation, differentiation, and survival. Among the activated downstream pathways from RAS is the mitogen-activated protein kinase (MAPK). MEK1 (also known as MAP2K1), a serine-threonine kinase, affects the regulation of growth-regulating proteins. KRAS mutations are the most common driver alterations, found in up to 25% of all patients with adenocarcinoma ^1^. Targeting KRAS itself has been challenging, primarily because KRAS activates multiple downstream effectors, including MEK, among many others. MEK1 inhibitors in the setting of RAS mutations are currently undergoing testing ^37– 39^. Tao *et al.* recently demonstrated that KRAS mutations can drive increased expression of cyclin-dependent kinase 4 (CDK4) and cyclin D1, facilitating cell proliferation and thus tumorigenesis ^40^. Additionally, a synthetic interaction between KRAS and CDK4 in animal models has proven to be lethal ^41^. While there is no specific targeted therapy for KRAS mutations, pre-clinical data suggest that the MEK inhibitor trametinib in combination with a CDK4/6 inhibitor (palbociclib) has significant anti-KRAS-mutant NSCLC activity ^40^.

### ROS1 mutation

The *ROS1* proto-oncogene receptor tyrosine kinase (ROS1) is activated by chromosomal rearrangement, which leads to the fusion of a portion of ROS1 that includes the entire tyrosine kinase domain with 1 of 12 different partner proteins ^42^ The ROS1 fusion kinases are then activated and result in cellular transformation. A total of 1% of patients with NSCLC have ROS1 rearrangements, and they are commonly found in never-smokers with histologic features of adenocarcinoma ^43^. The kinase domains of ALK and ROS1 share 77% amino acid identity within the ATP-binding sites. Crizotinib binds with high affinity to both ALK and ROS1 ^42, 44, 45^. Shaw and colleagues noted that crizotinib showed marked antitumor activity in patients with advanced ROS1-rearranged NSCLC ^46^. In March 2016, the FDA approved crizotinib for the treatment of patients with metastatic NSCLC whose tumors are ROS1 positive.

### MET mutation

The *MET* receptor tyrosine kinase is a known oncogene, with a somatic mutation frequency of 8.3% in lung adenocarcinoma and 2% in lung squamous cell carcinoma ^47^. Unlike activating *EGFR* mutations that occur primarily in the tyrosine kinase domain, *MET* mutations are distributed across all domains of the gene ^48^. MET can be activated as a primary oncogenic driver in NSCLC in at least two main ways: high-level *MET* amplification and *MET* exon 14 alterations. *MET* exon 14 skipping events occur in both adenocarcinoma and squamous cell carcinoma histology and are found across all smoking histories and histologic types. *MET* exon 14 skipping results from somatic mutations in the introns of *MET and* leads to an alternatively spliced transcript of MET ^49, 50^. The alternatively spliced MET receptor exhibits decreased ubiquitination and delayed downregulation, leading to prolonged activation of MET and MAPK ^49^. Overall, reports of *MET* exon 14 skipping have ranged from 1.5% to 6% of NSCLC ^49, 51– 54^. Patients harboring *MET* amplification, *MET* exon 14 alteration, and the combination of both have shown favorable response to MET tyrosine kinase inhibitors such as crizotinib in multiple case series, and these drugs continue to be a topic of ongoing investigation ^54– 58^.

### BRAF mutation

BRAF mutations act as an oncogenic driver via the MAPK pathway in NSCLC. The most common of these mutations, BRAF V600E (Val600Glu), is observed in 1 to 2% of lung adenocarcinomas ^3, 59– 61^. Despite unclear prognostic implications of BRAF V600E mutation, several studies have associated BRAF V600E with poor outcomes and lower response rates to platinum-based chemotherapy in patients with NSCLC compared with patients with NSCLC without BRAF mutations ^61, 62^. Dabrafenib, a selective BRAF inhibitor, demonstrated clinical activity with an overall confirmed response of 33% (95% CI: 23–45) and median PFS of 5.5 months in patients with previously treated NSCLC with BRAF V600E-mutant NSCLC ^63^. Additionally, BRAF plus MEK inhibition has demonstrated an increased overall response, PFS, and OS compared with BRAF-inhibitor monotherapy in patients with BRAF V600-mutant metastatic melanoma ^64– 66^. In a study of 57 patients previously treated with systemic chemotherapy for metastatic BRAF V600E-mutant NSCLC, the investigators showed an overall response of 63.2% (36 of 57, 95% CI: 49.3–75.6) with dabrafenib plus the MEK inhibitor trametinib ^67^. Investigations targeting BRAF V600E are ongoing.

## Anti-angiogenic agents

Angiogenesis is one of the hallmarks of cancer, and blockade of vascular endothelin growth factor receptor-2 (VEGFR-2) signaling inhibits the formation, proliferation, and migration of new blood vessels ^46^. Bevacizumab, a recombinant humanized monoclonal antibody against VEGF, when compared to carboplatin-paclitaxel first-line chemotherapy, resulted in a notable OS improvement in eligible patients with non-squamous NSCLC ^68^. The anti-VEGFR antibody ramucirumab is a human IgG1 monoclonal antibody that binds to the VEGFR-2 extracellular domain with high affinity, preventing the binding of all VEGF ligands and receptor activation ^69^. Ramucirumab improved PFS and OS when combined with docetaxel, irrespective of histology, and was approved by the FDA in NSCLC ^70^. In a multicenter, double-blind, randomized phase III trial, 1,253 patients with squamous and non-squamous NSCLC who progressed during or after a first-line platinum-based chemotherapy regimen were enrolled. Patients were randomized to receive docetaxel plus either ramucirumab or placebo. Median OS was 10.5 months (IQR: 5.1–21.2) among patients randomized to docetaxel plus ramucirumab and 9.1 months (4.2–18) for patients who received placebo plus docetaxel (hazard ratio [HR]: 0.86, 95% CI: 0.75−0.98, *P* = 0.023). In those given ramucirumab, median PFS was 4.5 months (IQR: 2.3–8.3), while the control group had a PFS of three months (IQR: 1.4–6.9) (HR: 0.76, 95% CI: 0.68–0.86, *P* <0.0001) ^71^.

## Immunotherapy in NSCLC

Therapies targeting the complex interaction between tumors and the immune system represent the largest recent breakthrough in cancer therapy. Immunotherapy is a particularly attractive option, as it is independent of the cancer cell mutational status and has been proven to be efficacious with a more acceptable side effect profile. Therapies targeting inhibitory checkpoint molecules including cytotoxic T-lymphocyte-associated protein 4 (CTLA4), programmed death 1 (PD-1), and programmed death ligand 1 (PD-L1) have all demonstrated clinical efficacy. CTLA4 expressed on T cells primarily regulates the extent of the early stages of T-cell activation ^72^. CTLA4 was the first immune checkpoint receptor to be clinically targeted. The exact mechanism of CTLA4 action is debatable, but its major role is thought to be in the down-modulation of helper T-cell activity and the enhancement of regulatory T-cell immunosuppressive activity ^72^. Ipilimumab was the first immunotherapeutic to show survival benefit in metastatic melanoma and was approved by the FDA in 2010 for the treatment of advanced melanoma ^73^. Therapies directed to other immune checkpoint receptors such as PD-1 and PD-L1 have emerged as promising targets. The PD-1 and B7-1 (also known as CD8) receptors, expressed on activated T cells, are connected by ligands PD-L1 and PD-L2, which are expressed by tumor cells and tumor-infiltrating lymphocytes ^74– 76^. Tumor PD-L1 expression is common in NSCLC. Indeed, in a study of 436 patients with NSCLC, PD-L1 expression was detectable in tumor cells in 34.5% (88/256) of patients with adenocarcinoma and 34% (61/180) of patients with squamous cell carcinoma ^77^. PD-1 interaction with both PD-L1 and PD-L2 ligands results in tumor immune recognition and elimination escape by inhibiting T-cell activation. The monoclonal antibody nivolumab directed to the PD-1 receptor has shown activity across NSCLCs with various histologic features. In a randomized trial of 272 patients receiving nivolumab versus docetaxel, the median OS was 9.2 months (95% CI: 7.3–13.3) versus 6.0 months (95% CI: 5.1–7.3), respectively ^78^. The 1-year OS rate was 42% (95% CI: 34–50) with nivolumab versus 24% (95% CI: 17–31) with docetaxel, while the response rate was 20% with nivolumab versus 9% with docetaxel ( *P* = 0.008). Nivolumab resulted in a median PFS of 3.5 months, whereas docetaxel demonstrated a median PFS of 2.8 months (HR for death or disease progression: 0.62, 95% CI: 0.47–0.81, *P* <0.001).

There was a 7% rate of grade 3 or 4 adverse events in the nivolumab group versus 55% in the docetaxel group. In March 2015, nivolumab was approved by the FDA for the management of squamous NSCLC. This was followed by FDA approval of nivolumab for the treatment of non-squamous NSCLC. In October 2015, Borghaei *et al.* showed that the median OS was 12.2 months (95% CI: 9.7–15.0) among 292 patients in the nivolumab group versus 9.4 months (95% CI: 8.1–10.7) among 290 patients in the docetaxel group (HR for death: 0.73, 96% CI: 0.59–0.89, *P* = 0.002). At 1 year, the OS rate was 51% with nivolumab versus 39% with docetaxel. At 18 months, the OS rate was 39% (95% CI: 34–45) with nivolumab versus 23% (95% CI: 19–28) with docetaxel ^79^.

In October 2015, another PD-1 receptor-directed monoclonal antibody, pembrolizumab, was approved by the FDA as a second-line treatment in NSCLC. The clinical efficacy of nivolumab and pembrolizumab was found to be independent of histology. Greater benefit was seen in smokers and in patients with PD-L1-positive expression ^78^. Although the clinical value of targeting the PD-L1-PD-1 pathway has been established, the POPLAR study evaluated the blockade of PD-L1–B7-1 interactions in addition to PD-L1-PD-1 blockade ^77^. In a multicenter, open-label, phase II, randomized controlled trial by Fehrenbacher *et al.*, the investigators evaluated the efficacy of atezolizumab versus docetaxel for patients with previously treated NSCLC (POPLAR trial) ^80^. Atezolizumab is a humanized IgG1 monoclonal anti-PD-L1 antibody which blocks PD-L1-PD-1 and PD-L1-B7-1 interactions, resulting in the restoration of anti-tumor T-cell activity and enhanced T-cell priming. OS in the intention-to-treat population was 12.6 months (95% CI: 9.7–16.4) for atezolizumab versus 9.7 months (95% CI: 8.6–12.0) for docetaxel (HR: 0.73, 95% CI: 0.53–0.99, *P* = 0.04). The FDA approved atezolizumab in October 2016 for the treatment of patients with metastatic NSCLC whose disease progressed during or following platinum-containing chemotherapy. Several trials comparing anti-PD-1 and anti-PD-L1 agents, individually and in combination with a cytotoxic T-lymphocyte-associated protein 4 inhibitor (ipilimumab), with platinum-based combination regimens as first-line therapy are ongoing ^81– 83^.

## Toxicities in the treatment of NSCLC

Although targeted therapy agents are directed against tumor cells, they are still associated with toxicities affecting multiple organs. Overall, the most common side effects of tyrosine kinase inhibitors and ALK inhibitors involve the gastrointestinal (43–60%), ophthalmic (55–62%), hematologic (5–10%), and cardiovascular (10%) systems ^84^. A total of 5% of patients discontinued crizotinib use due to toxicity ^85^. Liver toxicity is more frequent with gefitinib, and diarrhea and skin toxicity are frequent with afatinib. The most common adverse effects observed with ramucirumab were neutropenia (49%), febrile neutropenia (16%), and fatigue (16%). The number of deaths from adverse events was 5% ^86^.

The toxicities of immunotherapies in lung cancer are less frequent. The most commonly observed immune-related adverse effects of nivolumab involved the skin, gastrointestinal tract, and kidneys ^71^. All adverse effects including pneumonitis were managed effectively with corticosteroids or infliximab and did not result in mortality. Pembrolizumab has a similar safety profile, but one study demonstrated a <1% incidence of mortality from pneumonitis ^84^.

## Radiotherapy

Stereotactic ablative radiotherapy (SABR) has become a treatment option for early stage node-negative NSCLC patients who are not operative candidates or refuse surgical options ^87^. Despite its high tumor control rate and favorable toxicity profile relative to other surgical and non-surgical options, SABR use has been limited primarily to early stage disease ^87– 90^. Limited data from two pooled studies demonstrated the potential role for SABR in T1–2aN0M0 inoperable NSCLC ^91^. Patients were randomized to SABR (31 patients) versus lobectomy (27 patients). The estimated OS at three years was 95% (95% CI: 85–100) in the SABR group compared with 79% (95% CI: 64–97) in the surgery group (HR: 0.14, 95% CI: 0.017–1.190, log-rank *P* = 0.037). The SABR group had a recurrence-free survival at three years of 86% (95% CI: 74–100), while the surgery group’s was 80% (95% CI: 65–97). In addition to radiotherapy’s role in stage I NSCLC, new studies have examined its role in combination with chemotherapy in the management of stage IV NSCLC and have shown improved survival when radiotherapy was applied to the primary tumor. From a total of 274 patients in a study by Su *et al.*, 183 had non-oligometastatic disease. Patients with oligometastatic disease who received radiation to primary tumor had better OS ( *P* <0.001). For non-oligometastatic patients, multivariate analysis showed that receiving ≥63 Gy radiation, having a gross tumor volume of <146 cm ^3^, having response to chemotherapy, and having stable or increased post-treatment KPS independently predicted better OS ( *P* = 0.018, *P* = 0.014, *P* = 0.014, and *P* = 0.001, respectively). Among patients with greater primary tumor volume, high radiation dose remained of benefit for OS ^92^. Radiotherapy has also shown benefit in the setting of CNS involvement. Depending on the volume and number of brain metastases, stereotactic radiation is often preferred to whole-brain radiation therapy (WBRT), when feasible, mainly due to fewer neurological side effects. In patients with EGFR-mutant NSCLC with brain metastasis, a higher response rate is noted with WBRT. Additionally, WBRT improves the duration of intracranial disease control compared to tyrosine kinase inhibitors or stereotactic radiosurgery. Despite these results, many practitioners and patients avoid WBRT to prevent the hair loss, fatigue, and neurocognitive sequelae of WBRT ^93^. In patients with oligo-progressive disease on EGFR tyrosine kinase inhibitor monotherapy, local radiotherapy or surgical resection is recommended with continuation of the EGFR tyrosine kinase inhibitor on a case-by-case basis ^93– 95^.

## Surgical therapy

Various techniques are being investigated to make surgical resection less invasive. Sublobar resection and single-port video-assisted thoracoscopic surgery (VATS) have shown promising results ^96^. In their study of 121 patients (88% adenocarcinoma, 76% stage I), Hsu *et al.* used single-port VATS segmentectomies and lobectomies and concluded that resection was both safe and feasible in a multi-institutional retrospective analysis. They identified patient age of 60 years or more, male sex, and tumor size greater than 3 cm as unfavorable preoperative outcome predictors. The results of this study remain to be validated in a randomized controlled prospective study ^96^. Altorki and colleagues compared patients treated with sublobar resection to patients treated with lobectomy in stage I NSCLC and showed no significant 10-year survival difference among the two groups ^97^. Additionally, Burdett *et al.* showed that patients with stage IB, II, or III NSCLC who received chemotherapy following surgery (with or without radiotherapy) had longer survival than those who had surgery without chemotherapy (with or without radiotherapy) ^97^. The role of surgery has also changed secondary to the implementation of lung cancer screening within and outside the USA.

Early cancer detection using low-dose CT screening showed a 20% reduction in lung cancer mortality in the National Lung Screening Trial (NLST) ^98, 99^. Major goals of surgical participation in lung cancer screening programs were defined and established in Europe by the European Society of Thoracic Surgeons ^100^. These goals include optimization of the management of screen-detected nodules, reduction of false-positive rates of surgical biopsies, reduction of surgical incision-related trauma, implementation of national or international risk assessment guidelines, implementation of a smoking cessation policy, and active education of primary care physicians towards lung cancer screening programs. Additionally, it is important to emphasize the role of surgical resection in the diagnosis of specific histologic types of lung cancer as per the 2015 World Health Organization (WHO) classification of lung tumors and the eighth edition of the TNM classification. It is emphasized that in tumors such as adenocarcinoma *in situ* (AIS) and minimally invasive adenocarcinoma (MIA), large cell carcinoma, adenosquamous carcinoma, and pleomorphic carcinoma, the diagnosis cannot be made without complete evaluation of the entire tumor histologically ^101, 102^. Additionally, although the role of surgery in stage IIIA N2 disease remains controversial, in a meta-analysis of 868 patients from six trials, McElnay *et al.* showed that the HR comparing patients randomized to surgery after chemotherapy was 1.01 (95% CI: 0.82–1.23, *P* = 0.954), whereas patients randomized to surgery after chemoradiotherapy demonstrated a HR of 0.87 (95% CI: 0.75–1.01, *P* = 0.068). The overall HR of all pooled trials was 0.92 (95% CI: 0.81–1.03, *P* = 0.157) ^103^. The authors concluded that surgery should be considered as part of the multimodal treatment for patients with resectable lung cancer and ipsilateral mediastinal nodal disease. In the trimodality regimen, a 13% relative improvement in OS was noted with surgical results. Resection for patients with oligometastatic disease represents a relatively new concept in thoracic surgery. In patients with oligometastatic disease, local treatment for distant metastases and curative-intent pulmonary resection may lead to longer survival but requires an optimal method of patient selection. Additional trials are required to determine the ideal treatment plan and long-term follow up in this patient population ^104– 106^.

## Conclusion

The identification of driver mutations has led to a new era of personalized targeted therapy for NSCLC. This has created hope for a disease that carries a high morbidity and mortality. Cutting-edge interrogation of the tumor genome has provided a unique opportunity to investigate potentially targetable molecular aberrations in tumor suppressor genes and oncogenes. Paired with effective targeted therapies, these advances can help improve NSCLC outcomes. However, resistance to targeted therapies and the ongoing demand for better understanding of driver genomic aberrations requires continued and relentless effort. Studies of novel checkpoint inhibitors and numerous trials exploring the role of immunotherapy are ongoing, although there have been promising results with favorable toxicity profiles using immunotherapeutic agents. Combination therapeutics with chemotherapy, immunotherapy, and/or stereotactic ablative therapy need to be studied further.
